# Effect of Voltage and concentration of polyetherimide on surface morphology and corrosion properties of AZ91D by electro-spin coating

**DOI:** 10.1016/j.heliyon.2024.e31884

**Published:** 2024-05-24

**Authors:** S. David Blessley, P. Narayanasamy, P. Balasundar, B. Balavairavan

**Affiliations:** a Department of Mechanical Engineering, Francis Xavier Engineering College, Tirunelveli, Tamilnadu, India; b Department of Mechanical Engineering, Kamaraj College of Engineering and Technology, Near Virudhunagar, Madurai, Tamilnadu, India; c Department of Mechatronics Engineering, Kamaraj College of Engineering and Technology, Near Virudhunagar, Madurai, Tamilnadu, India

**Keywords:** AZ91D, PEI, Electro spin coating, Microstructure, AFM, Corrosion

## Abstract

Magnesium alloys, particularly AZ91D, exhibit promising mechanical properties but are susceptible to corrosion, limiting their widespread industrial applications. This manuscript investigates the impact of voltage and concentration of Polyetherimide (PEI) on surface morphology and corrosion characteristics of AZ91D through electro-spin coating. PEI, known for its high strength and corrosion resistance, is applied using an eco-friendly electro-spin coating method. The study optimizes polymer concentration and applied voltage to enhance the anticorrosive properties of AZ91D. Atomic force microscopy (AFM) and scanning electron microscopy (SEM) reveal the morphological alterations, while electrochemical corrosion tests provide insights into the corrosion resistance. The results show that a moderate PEI concentration (15 %) at 14 kV voltage exhibits the most favorable corrosion resistance, emphasizing the need to optimize both parameters for enhanced protection of AZ91D against corrosion. The results contribute to developing economical and effective corrosion protection techniques for magnesium alloys, mainly for automotive applications.

## 1 Introduction

Magnesium alloys possess numerous advantages over aluminum alloys and steels due to their high specific strength, low density, excellent castability and weldability, resistance to aging, superior electrical and thermal conductivity, recyclability, and widespread availability [1,2]. Magnesium alloys, particularly AZ91D, possess promising mechanical properties but are susceptible to corrosion, limiting their general industrial applications [[3], [4], [5]]. Mallick et al. [6] highlighted the complex nature of magnesium corrosion, influenced by various factors like microstructure, impurities, and environmental conditions. Many researchers have considered using the alloying technique to enhance Mg's corrosion resistance. However, alloying is a solution that is both difficult and expensive to implement [7].

The surface modification techniques have been recognized because simple alloying cannot protect magnesium alloys from extreme conditions. These include both chemical and physical anticorrosive coatings, which improve the material's surface properties to be used in a broad spectrum of applications [[8], [9], [10], [11]]. CVD, PVD, and laser coating techniques are selected for applying coatings to increase corrosion resistance [[12], [13], [14]]. Industries commonly employ curtain coating, spray coating, dip coating, and spin coating procedures for applying coatings [15]. The polymer is dissolved in a suitable solvent, applied to the substrate, and let to dry in all these processes. The choice of solvent, its concentration, application method, and drying process significantly impact the characteristics of the substrate, hence influencing the morphology and corrosion resistance of the metal coating [16,17]. Polymer coating is a simple, efficient, and economical way to increase the corrosion resistance of Magnesium and its alloys. The application of polymers as a coating on biodegradable magnesium alloys revealed higher corrosion resistance, improved cytocompatibility, and osteogenesis potential for use in orthopedic implant applications [[18], [19], [20]].

Electro-spin coating attracts attention due to its uniform thickness, low polymer requirement, and fast solvent evaporation. It could also improve corrosion resistance. Electro-spin coatings are gaining popularity due to their ultra-thin and homogeneous polymer coatings. The electro-spin coating method has more advantages and is eco-friendly for polymer coating [21]. Studies by Vladu et al. [22] and Rivero et al. [23] demonstrated the efficacy of electro-spin coating in improving corrosion resistance on various metallic substrates.

Polyetherimide (PEI) possesses several properties, including high strength and rigidity even at high temperatures, resistance to heat and corrosion over an extended period, dimensional stability, and good electrical properties [24]. PEI is an amorphous high-temperature resin that, like other amorphous high-temperature resins, has excellent dimensional stability and is naturally flame retardant. Several studies have explored its potential to enhance the corrosion resistance of metal substrates. Jose et al. [25] highlighted the effectiveness of PEI coatings in improving the anticorrosion properties of metals due to their barrier properties and ability to hinder corrosive species penetration. Yet the drawback is that PEO coatings are insufficient in providing long-term protection for magnesium alloys because of their porosity and minor fractures [21]. Corrosion prevention of AZ31 alloy with PEI coatings utilizing the dip coating technique under different pretreated substrates significantly improved the corrosion rate.

J. Ali Syed et al. [26] developed multilayer PANI-PAA/PEI composite coatings for 316 stainless steel corrosion prevention using alternative deposition. Spin coating and heating removed leftover water, increasing thickness linearly with layer number. In 3.5 % NaCl solution, PANI-PAA composite with PEI and multilayer structure synergistically improved corrosion resistance. An optimum layer number of 20 formed an interfacial oxide layer and extended corrosive ion diffusion, providing the highest corrosion protection. PANI-PAA composite and PANI-PAA/PEI multilayer coating increased adhesion, uniformity, and passivation to prevent corrosion. Electrochemical experiments indicated strong protection efficiency and increased performance. The study showed that the PANI-PAA/PEI multilayer coating improved 316 stainless steel corrosion resistance.

The voltage applied during electro-spin coating is essential for determining the morphology and thickness of the deposited coatings. Studies by Şener et al. [27] and Okutun et al. [28] on other metal substrates revealed that varying voltage influences the structural characteristics of electrospun coatings. Additionally, the concentration of the polymer solution significantly affects the coating thickness and uniformity, as demonstrated by Bakar et al. [29] in their investigation of different substrates. While studies on the electro-spin coating of polymers on magnesium alloys are limited, research by Panahi et al. and Soujanya et al. [30,31] explored the use of electro-spin coating with a polymeric material to prevent corrosion from magnesium alloys, showing promising results in terms of improved corrosion resistance.

Research has employed SEM imaging to examine coating microstructure, pore size distribution, and surface roughness to determine their performance. Several studies show that SEM can show how curing temperature, solvent evaporation rate, and coating thickness affect polymer coating microstructure and morphology. The effects of cross-linking density on coating surface roughness and adherence have been studied using SEM to optimize production procedures [32].

AFM is useful for characterizing polymer covering nanoscale topography and mechanical properties. AFM scans coatings with a sharp tip on a cantilever to measure nanoscale attributes such as roughness, adhesion, stiffness, and elasticity. A better understanding of polymer coating structure-property correlations has helped design functional coatings [33].

SEM and AFM have improved surface coating characterization by revealing their micro- and nanoscale morphology, topography, and mechanical properties. These methods are crucial to the design, development, and optimization of polymer coatings for corrosion protection, biomedical implants, microelectronics, and aerospace materials.

The literature highlights Magnesium's suitability for automotive applications but notes challenges like high reactivity and limited corrosion resistance. Polymer coatings offer a cost-effective solution. Research focuses on enhancing the corrosion resistance of AZ91D alloy through electro-spin coating. By varying polymer concentration and applied voltage, the aim is to optimize parameters for improved anticorrosive properties. Scanning Electron Microscopy (SEM) and Atomic Force Microscopy (AFM) analyze morphology, while electrochemical corrosion tests assess resistance, emphasizing the critical role of both parameters.

## 2 Materials and methods

### 2.1 Specimen preparation and coating

**Fig. 1:**
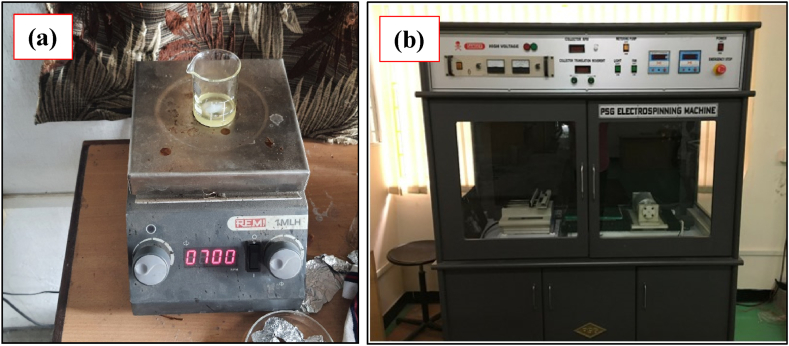
a) Magnetic Stirrer and b) Electrospinning Experimental Setup.

Commercial AZ91D alloy procured from Parshwamani Metals, Maharastra, measuring 25 mm × 25 mm x 4 mm, was used as the substrate, and the elemental composition was analyzed using an inductively coupled plasma atomic emission spectrometer (ICP), the results of which are detailed in Table 1. Before pretreatment, the samples underwent ultrasonic cleaning using acetone, ethanol, and distilled water. The substrate underwent pretreatment involving grinding with ascending grades of 500–2000, followed by acetone degreasing. Subsequently, a two-step treatment involving HF immersion and acetic acid dipping was conducted to remove impurities and enhance surface cleanliness. Solutions of PEI polymer in 1-Methyl 2 pyrrolidone (procured from Sigma Aldrich) at concentrations of 10 %, 15 %, and 20 % (w/v) were prepared by stirring PEI pellets in solvent using a magnetic stirrer as shown in Fig. 1 (a) at 60 °C and 700 rpm for 60 min. Electrospinning was performed using an experimental setup as depicted in Fig. 1 (b) with varying spinning voltages (11, 14, and 17 kV), solution concentrations, and specific parameters, including a needle tip to collector distance of 16 cm, solution flow rate of 0.2 mL/h, temperature of 25 °C, optimal relative humidity value 40 % and current of 20 μA.

**Table 1:** Elemental composition as per ICP.

Element	Al	Mn	Zn	Si	Cu	Fe	Ni	Mg
In wt. %	8.3–9.7	0.15–0.5	0.36	0.11	0.0320	0.0054	0.0020	Remaining

### 2.2 Scanning electron microscopy (SEM)

The surface morphology of the coated samples was studied using scanning electron microscopy (SEM). The samples underwent gold sputtering using an auto fine coater before SEM observations.

### 2.3 Atomic force microscopy (AFM)

The atomic force microscopy (AFM) technique was employed to assess the surface topography and roughness of the PEI coatings. AFM analysis was done using a Veeco Dimension Icon instrument working in contact mode in an ambient environment. NanoScope Analysis software was utilized to characterize the images.

### 2.4 Electrochemical corrosion test

A three-electrode system was used to study the corrosion behavior of PEI-coated AZ91D. The electrolytes used were 3.5 % NaCl. Pt was used as the counter electrode, Ag/AgCl was used as the reference electrode, and PEI-coated AZ91D was used as the working electrode. The contours representing potentiodynamic polarization (PDP) were acquired at a scan rate of 10 mV/s. The Icorr and Ecorr were derived from the experimental potentiodynamic curves utilizing the software CorrView to obtain the fitting parameters.

## 3 Results and discussion

### 3.1 Morphology study

**Fig. 2:**
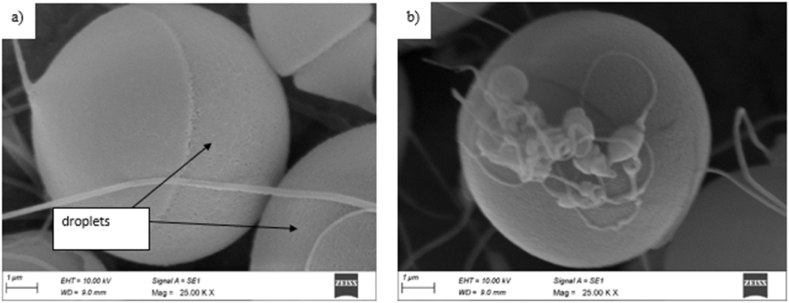
SEM image of AZ91D with a) 7.5 % PEI (w/v) b) 25 % PEI (w/v).

The morphologies of the electrospun fibers were observed under a scanning electron microscope, shown in Fig. 2(a and b). PEI solutions of a concentration less than 7.5 % did not produce any fibers; instead, only spheres or droplets were formed, as shown in Fig. 2(a). In PEI concentrations above 10 %, a mixture of round and spindle-like beads lodged in the fibers (flat and round) is observed. Such structures are desired for this study where tiny fibers may give the needed broad surface area, and relatively big beads may operate as repositories, allowing for targeted controlled release, especially for drug delivery, tissue engineering, and automobile applications. Table 2 shows the SEM photographs of different electrospun products of AZ91D with varying voltages and concentrations. From Tables 2 and it was observed that the diameter of the fiber increases with an increase in the concentration of PEI. As the concentration of PEI increases, the viscosity increases; hence, the thickness impacts due to the lower outflow of a more viscous solution. Table 2 shows that the PEI 20 %(w/v) solutions had higher viscosities when compared to PEI 10 & 15 % (w/v), producing thicker coatings under each category. Electrospinning of PEI concentrations above 25 % failed to yield fibers, as the solvent evaporated before forming a jet. As a result, the continuous flow of the solution to the capillary tip was blocked, as mentioned in Fig. 2 b.

**Table 2:** SEM images of AZ91D with PEI concentrations 10 %, 15 % and 20 % (w/v) and voltages 11 kV, 14 kV & 17 kV.

PEI Concentration (w/v)	Voltage (kV)		
	11 kV	14 kV	17 kV
PEI 10 %			
PEI 15 %			
PEI 20 %			

Table 2 demonstrates a positive correlation between voltage and Average Fiber diameter, as increasing the voltage from 11 kV to 14 kV increases the Average Fiber diameter. However, it is observed that the Average Fiber Diameter (AFD) exhibits a reduction when the applied voltage exceeds 17 kV. As the magnitude of the applied voltage is augmented, there is a corresponding augmentation in the velocity of the jet, leading to the rapid removal of solution from the capillary tip. As the size of the droplet on the spinneret decreases, it undergoes oscillation and develops a non-symmetric pattern. The jet is generated from the interface between the liquid surface and the tip, forming beads. The fiber diameter distribution (AFD) analysis revealed that the fibers produced by electrospinning at 17 kV had a reduced average diameter compared to those obtained at lower applied voltages. The Average Fiber Diameter (AFD) tends to decrease as the applied voltage increases due to the stretching effect of the fibers comprising the Polyetherimide (PEI) concentration.

The observed morphological variations in electrospun fibers can be attributed to the Polyetherimide (PEI) concentration and applied voltage. The absence of fibers at PEI concentrations below 7.5 % and the formation of only spheres or droplets suggest a critical concentration threshold required for fiber formation. This aligns with the concept of the critical entanglement concentration in polymer solutions, where below this concentration, polymer chains are insufficiently entangled to form stable fibers. Conversely, at higher PEI concentrations, a mixture of fibers and beads is observed, indicating a balance between solution viscosity, jet stability, and electrostatic forces during electrospinning. The correlation between PEI concentration and fiber diameter can be explained by the impact of solution viscosity on fiber morphology. Higher PEI concentrations result in increased solution viscosity, leading to thicker fibers due to reduced outflow rates. This aligns with the concept of solution viscosity affecting jet stability and fiber diameter during electrospinning.

### 3.2 Atomic force microscopy

The PEI coatings' surface topography and roughness were analyzed using Atomic Force Microscopy (AFM) with a Veeco Dimension Icon in contact mode under ambient air conditions. Table 3 depicts 2D AFM topographic images of PEI coatings over AZ91D alloy in scan areas of 10 μm × 10 μm. The 2D images show that the alloy samples have coatings free from fractures or flaws. It is also shown that the percentage of PEI content affects the morphology of the coatings. The absence of fractures or flaws indicates the effectiveness of the coating process in providing a continuous and intact layer of PEI on the alloy surface. The surface of the coating with 10 % PEI is smoother than the surfaces of the other coatings, suggesting superior uniformity. Three roughness parameters were employed to quantitatively assess the surface attributes of PEI coatings. These parameters encompass the average roughness (R_a_), root mean squared roughness (R_q_), and the most significant vertical height between the highest peak and lowest valley (R_z_). From Table 4, it is evident that the electrospun fibers are at the nano level, and higher the PEI content, greater the average fiber diameter, as depicted in the SEM images (Table 2).

**Table 3:** 2D Atomic Force Microscopy (AFM) images of AZ91D with PEI concentrations 10 %, 15 % and 20 % (w/v) and voltages 11 kV, 14 kV & 17 kV.

PEI Concentration (w/v)	Voltage (kV)		
	11 kV	14 kV	17 kV
PEI 10 %			
PEI 15 %			
PEI 20 %

**Table 4:** 3D Atomic Force Microscopy (AFM) images of AZ91D with PEI concentrations 10 %, 15 % and 20 % (w/v) and voltages 11 kV, 14 kV & 17 kV.

PEI Concentration (w/v)	Voltage (kV)		
	11 kV	14 kV	17 kV
PEI 10 %			
PEI 15 %			
PEI 20 %			

The AFM analysis reveals the influence of PEI concentration and applied voltage on the surface roughness of coatings. The observed increase in roughness with higher PEI concentrations can be attributed to the presence of larger polymer beads and thicker fibers, which create a more textured surface. Similarly, the decrease in roughness with increasing applied voltage suggests a transition towards finer fibers and smoother surfaces due to enhanced jet stretching and decreased bead formation at higher voltages.

The root-mean-square (RMS) roughness (R_q_) is found to be 47.95, 59.52, and 54.08 nm for the PEI 10 %, PEI 15 % & PEI 20 % coatings, respectively, at 11 kV of applied voltage. As the applied voltage increases, the RMS values tend to decrease, as depicted in Table 5. This is due to thinner and finer polymer fibers forming, which can result in a higher surface area and potentially smoother surfaces.

**Table 5:** Root mean square and average roughness value.

Concentration	Voltage	RMS Value (R_q_)	Average Roughness (R_z)_ in nm
PEI 10 %	11 kV	47.95	900
	14 kV	22.83	256
	17 kV	11.29	608
PEI 15 %	11 kV	59.52	776
	14 kV	47.11	625
	17 kV	21.05	1070
PEI 20 %	11 kV	54.08	1070
	14 kV	41.11	263
	17 kV	19.81	1589

The Peak-to-Peak height R_z_ distribution of PEI coatings deposited at different voltages can also be discussed using AFM. The average R_z_ values of 10 % PEI, 15 % PEI & 20 % PEI at 11 kV are found to be 900 nm, 776 nm & 1070 nm, respectively. When the voltage applied varies from 11 kV to 14 kV, the R_z_ values are approximately 256 nm, 625 nm & 263 nm, respectively. Further increasing the voltage from 14 kV to 17 kV, the average R_z_ value ranges are 608 nm, 1070 nm, and 1589 nm, respectively. Therefore, when the applied voltage varies from 11 kV to 14 kV, the PEI coatings correspond to lower peak-to-peak height. Skewness, S_sk_, is a dimensionless amplitude parameter of surface roughness that signifies the presence of “sharp” features on the surface. Table 6 shows the skewness value of PEI coatings of different concentrations at different voltages. It can be observed from the table that increasing the voltage tends to provide a symmetric distribution of fibers in the case of 10 % PEI & 15 % PEI, respectively, whereas increasing the voltage produces positive and negative skew in the case of 20 % PEI, which means the distribution is inhomogeneous and might promote the formation of porous structure.

**Table 6:** Skewness (S_sk_).

Skewness (S_sk_)	10 % PEI	15 % PEI	20 % PEI
11 kV	0.36	−0.22	0.14
14 kV	0.21	−0.13	−0.22
17 kV	−0.05	0.06	0.45

The variation in peak-to-peak height (R_z_) with PEI concentration and applied voltage further corroborates the impact of these parameters on surface topography. The observed skewness in fiber distribution at higher voltages and PEI concentrations indicates non-uniform fiber deposition, potentially leading to the formation of porous structures, as discussed in the literature.

### 3.3 Corrosion study

**Fig. 3:**
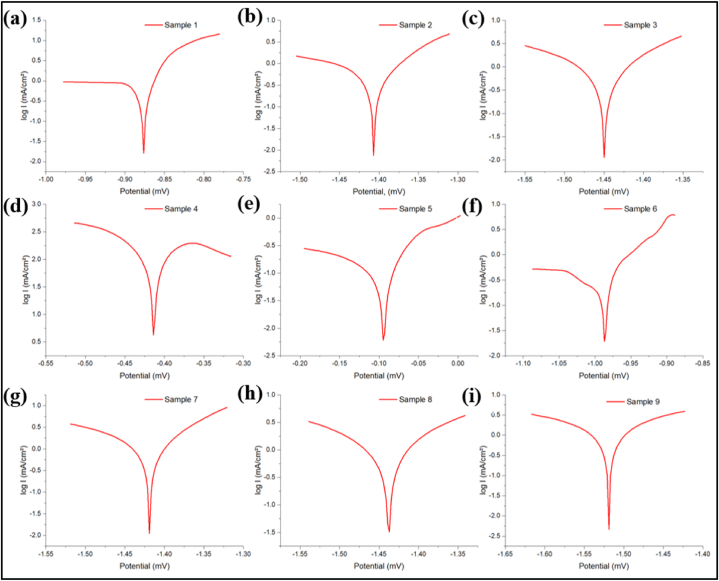
Tafel Plots of AZ91D under various PEI concentrations and voltage (a) 10 % with 11 kV, (b) 10 % with 14 kV, (c) 10 % with 17 kV, (d) 15 % with 11 kV, (e) 15 % with 14 kV, (f) 15 % with 17 kV, (g) 20 % with 11 kV, (h) 20 % with 14 kV, and (i) 20 % with 17 kV.

The section explores the impact of voltage and Polyetherimide (PEI) concentration on the surface morphology and corrosion properties of AZ91D using electro-spin coating. Fig. 3(a–i) shows the Tafel polarization curves of AZ91D coated with PEI polymer concentrations 10, 15, and 20 (w/v) at 11, 14 and 17 kV. The Ecorr, Icorr values and the corrosion rate obtained from the polarization curve are summarized in Table 7. It is observed that samples with 10 % PEI displayed a negative correlation when the voltage was increased from 11 kV to 17 kV (Fig. 3(a–c)). The Ecorr values of samples with 10 % PEI concentration were found to be −875.7, −1406.4, and −1449.5 mV under varying electro-spin voltages (Table 7). Increasing PEI concentration from 10 % to 15 % significantly enhanced corrosion resistance across all voltage levels compared to the former shown in Fig. 3(d–f). Sample 5 (PEI 15 %, 14 kV) demonstrated promising corrosion resistance, suggesting that a moderate PEI content contributes to a smoother coating surface. The E_corr_ values of sample 5 (PEI 15 %,14 kV) were found to be −94.3 mV, which is considerably more favorable in comparison to the E_corr_ values acquired from the diverse voltages of PEI 10 % and PEI 20 % concentration. On the other hand, the current density Icorr values of Sample 5 were obtained as 0.159 mA/cm^2^, which is the least of all the Icorr values at different polymer concentrations of varying voltage. This further confirms that moderate polymer content enhances corrosion resistance by providing barrier protection, chemical inhibition, and better adhesion that are compatible with the underlying Magnesium substrate.

**Table 7:** Results of electrochemical tests.

Details	PEI Concentration, w/v %	Voltage, kV	Ecorr, mV	I corr, mA/cm^2^	Corrosion rate, mm/Y
Sample 1	PEI 10	11	−875.7	0.866	19.972
Sample 2	PEI 10	14	−1406.4	0.6889	15.886
Sample 3	PEI 10	17	−1449.5	0.7074	16.314
Sample 4	PEI 15	11	−413.5	0.2439	5.6263
Sample 5	PEI 15	14	−94.3	0.159	3.6668
Sample 6	PEI 15	17	−986.5	0.3691	8.5112
Sample 7	PEI 20	11	−1418.7	1.4096	32.507
Sample 8	PEI 20	14	−1437.9	1.0339	23.842
Sample 9	PEI 20	17	−1519.6	0.9771	22.534

Samples with 20 % PEI concentration exhibited higher corrosion rates (Figure(g–i)). Increased polymer concentration and roughness parameters negatively impact corrosion resistance. A significant increase in Icorr values of 20 % PEI concentrated samples can be noted as the increase in supply voltage from 11 kV to 17 kV, as shown in Fig. 3(g–i) and Table 7. Higher voltages, significantly beyond 14 kV, exacerbated these effects, leading to decreased corrosion resistance [34]. The decrease in Ecorr values from −1418.7 to −1519.6 mV due to the voltage increase further confirms poor corrosion inhibition due to insufficient adhesion and limited penetration. When the polymer concentration is too high, it may lead to poor adhesion to the metal substrate.

Tafel plots indicated a potential compromise between high PEI concentration (above 15 %) and voltage (above 14 kV) regarding corrosion resistance. Moderate PEI concentrations and voltages generally improved corrosion resistance, emphasizing the optimization of PEI concentration and voltage for enhanced corrosion resistance in AZ91D coated with Polyetherimide using electro-spin coating.

The corrosion study elucidates the interplay between PEI concentration, applied voltage, and corrosion resistance of AZ91D coatings. The negative correlation between Ecorr values and applied voltage for samples with 10 % PEI concentration suggests a potential degradation of corrosion resistance at higher voltages (Fig. 3(a–c)). This could be attributed to increased bead formation and surface roughness, providing more sites for corrosion initiation and propagation. Conversely, samples with moderate PEI concentrations (15 %) demonstrate improved corrosion resistance, attributed to smoother coating surfaces and better adhesion. This aligns with the concept of barrier protection and chemical inhibition provided by polymer coatings, where an optimal balance between polymer content and surface roughness is crucial for corrosion resistance [35,36]. Elevated levels of polymer content and roughness characteristics have an adverse effect on corrosion resistance. An observable rise in Icorr values may be observed in 20 % concentrated samples of PEI as the supply voltage is increased from 11 kV to 17 kV (Fig. 3(g–i)). Increased voltages above 14 kV worsened these effects, reducing corrosion resistance. The decline in Ecorr readings resulting from the voltage increase provides more evidence of inadequate corrosion inhibition caused by inadequate adhesion and restricted penetration. This result concludes excessive polymer concentration might result in inadequate adherence to the metal substrate.

### 3.4 Microstructure analysis of corroded samples

**Fig. 4:**
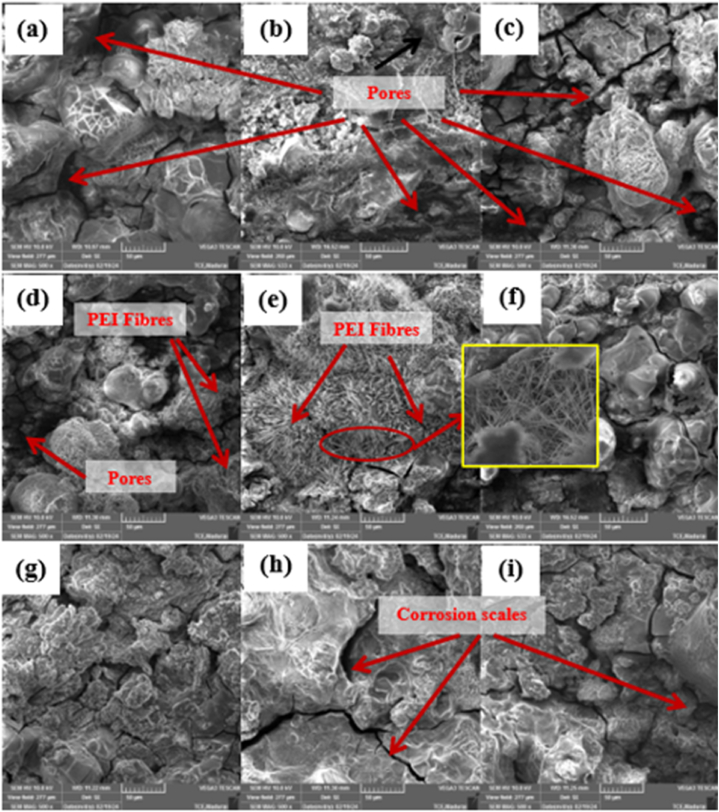
Microstructure analysis of corroded Samples of varying PEI concentrations and voltage (a) 10 % with 11 kV, (b) 10 % with 14 kV, (c) 10 % with 17 kV, (d) 15 % with 11 kV, (e) 15 % with 14 kV, (f) 15 % with 17 kV, (g) 20 % with 11 kV (h) 20 % with 14 kV, and (i) 20 % with 17 kV.

Following the electrochemical corrosion test, the surface morphologies of all the samples coated with PEI were analyzed using SEM (Fig. 4(a–i). Upon examining images in Fig. 4(a–c) and Fig. 4(g–i), it is evident that most coated surfaces with 10 % and 20 % PEI exhibit unstable pits, cracks, and cavities [37]. Excessively accumulated salt residues on the coated surfaces generally degraded the coating's morphology when the PEI concentration was 15 %, especially at 14 kV. The built-up salt residues are referred to as corrosion products. Corrosion product generation is significant in Fig. 4(d–f). More salt residues are deposited over the fibers of specimens with 15 % PEI content (Fig. 4(e)). Therefore, this sample blocks the transmission of reactive ions to the substrate, resulting in improved anticorrosion characteristics.

(1) **Anode Reaction (Oxidation):** Mg→Mg^2+^+2e^−^

Magnesium (Mg) undergoes oxidation at the anode, releasing electrons into the metal. The released electrons contribute to the electrical current, forming magnesium ions.

(2) **Cathode Reaction (Reduction):** 2H_2_O+2e^−^→H_2_(*g*)+2OH^−^

At the cathode, water (H₂O) is reduced by accepting electrons, producing hydrogen gas (H₂) and hydroxide ions (*OH*−).

The overall electrochemical corrosion reaction for Mg in a chloride environment can be represented by combining the anode and cathode reactions:

(3) Mg+2H_2_O→Mg^2+^+H_2_(*g*)+2OH^−^

The presence of corrosion pits and pores in the corroded samples of PEI 10 % concentration is due to the formation of porous structures, as shown in Fig. 4(a–c), which often exhibit a greater surface area than non-porous materials. This increased surface area provides more sites for corrosion initiation and propagation. Inadequate adhesion can result in the formation of defects or scales in the case of a PEI concentration of 20 % over the protective polymer coating, exposing the metal to corrosive agents and promoting corrosion (Fig. 4 g-i). Also, extremely high polymer concentrations may hinder the penetration of the polymer into the substrate's microstructure. This limitation in penetration can leave certain areas of the metal unprotected, making them susceptible to corrosion.

## 4 Conclusions

In conclusion, this manuscript delves into the intricate relationship between voltage, Polyetherimide (PEI) concentration, and their combined impact on the surface morphology and corrosion properties of AZ91D using an eco-friendly electro-spin coating technique. The results reveal a moderate PEI concentration (15 %) at 14 kV voltage yields the most favorable corrosion resistance.

☐Concentrations above 10 % resulted in a combination of round and spindle-like beads within the fibers. A positive correlation was observed between voltage and average fiber diameter up to 17 kV. Beyond this voltage, the fiber diameter is reduced. Higher voltage increased jet velocity, causing rapid solution removal and leading to non-symmetric patterns and bead formation.
☐The percentage of PEI content affected coating morphology, with 10 % PEI producing a smoother surface, indicating better homogeneity. Higher PEI content led to increased fiber diameter and roughness parameters. Higher voltage decreased root mean squared roughness (R_q_), attributed to thinner and finer polymer fibers producing potentially smoother surfaces.
☐Lower voltages corresponded to lower R_z_ values, indicating smoother coatings. Increasing voltage beyond 14 kV resulted in increased R_z_ values, potentially promoting the formation of porous structures.
☐The skewness values indicated varying distributions of fibers. Higher voltages caused a shift towards positive and negative skewness in the case of 20 % PEI, suggesting an inhomogeneous distribution that might promote porous structure formation.
☐Corrosion results indicated that the 15 % PEI at 14 kV of applied voltage exhibited favorable resistance against corrosion, implying that a moderate PEI concentration contributes to a more even coating surface.
☐When exposed to corrosion agents, increasing PEI concentrations to 20 % of AZ91D results in poor adhesive qualities. Extremely high polymer concentrations may inhibit the polymer's entry into the substrate's microstructure.
☐ This research contributes valuable insights towards developing efficient and cost-effective corrosion protection methods for magnesium alloys, particularly in automotive applications.

## Ethical approval

Not Applicable.

## Funding

Not Applicable.

## Data availability statement

No data was used for the research described in the article.

## CRediT authorship contribution statement

**S. David Blessley:** Writing – original draft, Visualization, Validation, Software, Resources, Project administration, Methodology, Investigation, Funding acquisition, Formal analysis, Data curation. **P. Narayanasamy:** Writing – review & editing, Writing – original draft, Validation, Supervision, Investigation, Formal analysis, Data curation, Conceptualization. **P. Balasundar:** Writing – review & editing, Visualization, Validation, Investigation, Formal analysis. **B. Balavairavan:** Writing – review & editing, Validation, Methodology, Formal analysis.

## Declaration of competing interest

The authors declare that they have no known competing financial interests or personal relationships that could have appeared to influence the work reported in this paper.
